# Lack of Association between *JAK3* Gene Polymorphisms and Cardiovascular Disease in Spanish Patients with Rheumatoid Arthritis

**DOI:** 10.1155/2015/318364

**Published:** 2015-03-01

**Authors:** Mercedes García-Bermúdez, Raquel López-Mejías, Fernanda Genre, Santos Castañeda, Alfonso Corrales, Javier Llorca, Carlos González-Juanatey, Begoña Ubilla, José A. Miranda-Filloy, Trinitario Pina, Carmen Gómez-Vaquero, Luis Rodríguez-Rodríguez, Benjamín Fernández-Gutiérrez, Alejandro Balsa, Dora Pascual-Salcedo, Francisco J. López-Longo, Patricia Carreira, Ricardo Blanco, Javier Martín, Miguel A. González-Gay

**Affiliations:** ^1^Institute of Parasitología and Biomedicina López-Neyra, IPBLN-CSIC, 18016 Granada, Spain; ^2^Epidemiology, Genetics and Atherosclerosis Research Group on Systemic Inflammatory Disease, Rheumatology Division, Hospital Universitario Marqués de Valdecilla, IDIVAL, Avenida de Valdecilla s/n, 39008 Santander, Spain; ^3^Rheumatology Department, Hospital Universitario La Princesa, IIS-Princesa, 28006 Madrid, Spain; ^4^Department of Epidemiology and Computational Biology, School of Medicine, University of Cantabria, and CIBER Epidemiology and Public Health (CIBERESP), IDIVAL, 39011 Santander, Spain; ^5^Cardiology Division, Hospital Universitario Lucus Augusti, 27003 Lugo, Spain; ^6^Division of Rheumatology, Hospital Universitario Lucus Augusti, 27003 Lugo, Spain; ^7^Department of Rheumatology, Hospital Universitario Bellvitge, 08907 Barcelona, Spain; ^8^Department of Rheumatology, Hospital Clínico San Carlos, 28040 Madrid, Spain; ^9^Department of Rheumatology, Hospital Universitario La Paz, 28046 Madrid, Spain; ^10^Department of Rheumatology, Hospital General Universitario Gregorio Marañón, 28007 Madrid, Spain; ^11^Department of Rheumatology, Hospital Universitario 12 de Octubre, 28041 Madrid, Spain; ^12^Cardiovascular Pathophysiology and Genomics Research Unit, School of Physiology, Faculty of Health Sciences, University of the Witwatersrand, Johannesburg 2000, South Africa

## Abstract

Rheumatoid arthritis (RA) is a polygenic disease associated with accelerated atherosclerosis and increased cardiovascular (CV) mortality. JAK/STAT signalling pathway is involved in autoimmune diseases and in the atherosclerotic process. JAK3 is a highly promising target for immunomodulatory drugs and polymorphisms in *JAK3* gene have been associated with CV events in incident dialysis patients. Therefore, the aim of this study was to assess the potential role of *JAK3* polymorphisms in the development of CV disease in patients with RA. 2136 Spanish RA patients were genotyped for the rs3212780 and rs3212752 *JAK3* gene polymorphisms by TaqMan assays. Subclinical atherosclerosis was evaluated in 539 of these patients by carotid ultrasonography (US). No statistically significant differences were found when each polymorphism was assessed according to carotid intima-media thickness values and presence/absence of carotid plaques in RA, after adjusting the results for potential confounders. Moreover, no significant differences were obtained when RA patients were stratified according to the presence/absence of CV events after adjusting for potential confounders. In conclusion, our results do not confirm association between *JAK3* polymorphisms and CV disease in RA.

## 1. Introduction

Rheumatoid arthritis (RA) is a chronic inflammatory rheumatic disease associated with an increased risk for cardiovascular (CV) events and CV-related deaths compared with the general population [1]. Evidence indicates that RA is an independent risk factor for premature heart disease [2]. This process can be partly explained by traditional CV risk factors [3], magnitude, and severity of a chronic inflammatory response [4], and genetic factors located inside [4] and outside the Human Leukocyte Antigen (HLA) region [5–8].

Janus kinases (JAKs) play a pivotal role in cytokine receptor signalling since they phosphorylate and activate signal transducer and activator of transcription (STAT) proteins. Several of these JAK-controlled cytokine receptor pathways are intimately involved in the initiation and progression of RA disease pathogenesis, autoimmune type-1 diabetes, systemic lupus erythematosus, and other autoimmune diseases [9–11]. The JAK/STAT pathway is a widely expressed intracellular signal transduction pathway, fundamentally important for T lymphocyte differentiation and function [12, 13]. This is of particular relevance since CD4+ T helper type 1 (TH1) cells are believed to promote atherosclerotic lesions and acute coronary syndromes, while T helper type 2 (TH2) cells likely serve an inhibitory or modulatory role [14, 15]. Furthermore, this signalling pathway controls important inflammatory processes in vascular cells, and its activation is involved in atherosclerosis and hypertension [16, 17].

JAK3 is the only Jak family member that associates with just one cytokine receptor, the common *γ* (*γ*c) chain, which is exclusively used by the receptors for IL-2, IL-4, IL-7, IL-9, IL-15, and IL-21 [11]. Although JAK1, JAK2, and Tyk2 are expressed ubiquitously, JAK3 expression is restricted to hematopoietic lineage cells [18]. The genes encoding the JAK family members are located on three separate chromosomes. The* JAK1* and* JAK2* genes are located on human chromosomes 1p31.3 and 9p24. In contrast, the gene coding for JAK3 is located on human chromosome 19p13.1 [18].

Different genetic variants located in the* JAK3* gene have been associated with some inflammatory disorders including the development of CV events in incident dialysis patients [19]. Interestingly, tofacitinib, a molecule that inhibits JAK3 and JAK1 and to a lesser extent JAK2, has shown robust and sustained efficacy in patients with RA [20].

Taking into account all these considerations, the main purpose of this study was to determine, for the first time whether* JAK3* gene variants in RA patients are associated with the presence of subclinical atherosclerosis and CV events.

## 2. Patients and Methods

### 2.1. Patients and Study Protocol

A set of 2136 Spanish patients with RA were included in the present study. Blood samples were obtained from patients recruited from Hospital Lucus Augusti (Lugo), Hospital Marqués de Valdecilla (Santander), Hospital de Bellvitge (Barcelona), Hospital Clínico San Carlos, Hospital La Paz, Hospital La Princesa, Hospital Gregorio Marañón, and Hospital 12 de Octubre (Madrid). A subject's written consent was obtained in all the cases. The Ethics Committees of the corresponding hospitals approved the purpose of the work. All the patients fulfilled the 1987 American College of Rheumatology (ACR) and the 2010 classification criteria for RA [21, 22]. Patients were assessed for rs3212780 and rs3212752* JAK3* gene variants. In addition, carotid intima-media thickness (cIMT) and presence/absence of carotid plaques were determined by carotid ultrasonography (US) in 539 of these patients.

Information on the main demographic data, clinical characteristics, CV risk factors, and CV events of patients enrolled in the study is shown in Table 1. Additionally, the 18% of these patients had experienced CV events, 75.2% were women and the mean age at the time of disease onset was 50.8 years. Definitions of CV events and traditional CV risk factors were established as previously described [4].

### 2.2. Genotyping

DNA from patients was obtained from peripheral blood using standard methods.

The rs3212780 and rs3212752* JAK3* polymorphisms were genotyped with TaqMan predesigned single-nucleotide polymorphism genotyping assays in a 7900 HT Real-Time polymerase chain reaction (PCR) system, according to the conditions recommended by the manufacturer (Applied Biosystems, Foster City, CA, USA). Negative controls and duplicate samples were included to check the accuracy of genotyping.

### 2.3. Carotid US Examination

Measurement of the cIMT and presence/absence of carotid plaques were performed in 539 patients from Lugo and Santander by carotid US. Patients from Santander were assessed using a commercially available scanner, Mylab 70, Esaote (Genoa, Italy) equipped with 7–12 MHz linear transducer and the automated software guided technique radiofrequency—Quality Intima Media Thickness in real-time (QIMT, Esaote, Maastricht, Holland)—was used [23, 24]. Patients from Lugo were assessed using high-resolution B-mode ultrasound, Hewlett Packard SONOS 5500, with a 10 MHz linear transducer as previously reported [25]. cIMT was measured at the far wall of the right and left common carotid arteries, 10 mm from the carotid bifurcation, over the proximal 15 mm-long segment. cIMT was determined as the average of three measurements in each common carotid artery. The final cIMT was the largest average cIMT (left or right). The plaque criteria in the accessible extracranial carotid tree (common carotid artery, bulb, and internal carotid artery) were focal protrusion in the lumen at least cIMT >1.5 mm, protrusion at least 50% greater than the surrounding cIMT, or arterial lumen encroaching >0.5 mm, according to Mannheim consensus criteria [26]. The carotid plaques were counted in each territory and defined as no plaque, unilateral plaque, or bilateral plaques [23, 24, 27]. Agreement between the two US methods in patients with RA was previously reported [27]. Two experts with high experience and close collaboration in the assessment of subclinical atherosclerosis in RA from Santander (AC) and Lugo (CGJ) performed the studies.

### 2.4. Statistical Analysis

All genotype data were checked for deviation from Hardy-Weinberg equilibrium (HWE) using http://ihg.gsf.de/cgi-bin/hw/hwa1.pl.

cIMT values are displayed as mean and standard deviation (SD). The association between genotypes and alleles of each polymorphism and cIMT values was tested using unpaired *t*-test to compare between 2 groups and one-way analysis of variance (ANOVA) to compare among more than two groups. Comparisons of means was adjusted for sex, age at the time of US study, follow-up time and traditional CV risk factors (hypertension, diabetes mellitus, dyslipidemia, obesity, and smoking habit) as potential confounders using analysis of covariance (ANCOVA).

Differences in the genotypic and allelic frequencies of each polymorphism according to the presence/absence of carotid plaques and CV events were calculated by *χ* ^2^ or Fisher tests when necessary (expected values below 5). Strength of associations were estimated using odds ratios (OR) and 95% confidence intervals (CI). Results were adjusted for sex, age at the time of US study, and traditional CV risk factors (hypertension, diabetes mellitus, dyslipidemia, obesity, and smoking habit) by logistic regression.

Statistical significance was defined as *P* < 0.05. All analyses were performed with STATA statistical software 12/SE (Stata Corp., College Station, TX, USA).

## 3. Results

The* JAK3* rs3212780 and rs3212752 genotype distribution were in Hardy-Weinberg equilibrium.

As shown in Table 2, no differences were observed when genotype and allele frequencies from patients with or without CV events were compared for rs3212780 and rs3212752 gene variants. Results from an adjusted logistic regression model did not show statistically significant association between rs3212780 or rs3212752 gene polymorphisms and the risk of CV events.

As shown in Table 3, no statistically significant differences were found when each polymorphism was assessed according to the evaluation of the cIMT in RA patients, after adjusting the results for sex, age at the time of US study and traditional CV risk factors (hypertension, diabetes mellitus, dyslipidemia, obesity, and smoking habit) as potential confounders. Similarly, no statistically significant differences were detected when each polymorphism was evaluated according to the presence/absence of carotid plaques in RA, after adjusting the results for potential cofounder factors specified above (Table 3).

Taking into account the implication of* JAK3 *in inflammatory diseases and the relevant role of C-reactive protein (CRP) in inflammation, we assessed the potential association between* JAK3* polymorphisms and CRP levels in a representative subgroup of patients in whom CRP information was available. As shown in Table 4, we did not disclose a relationship between CRP levels neither with* JAK3* genotypes and alleles nor haplotypes.

Finally, we also evaluated whether there were differences in cIMT values and presence/absence of carotid plaques between patients positive and negative for rheumatoid factor (RF) and/or anti-cyclic citrullinated peptide antibodies (anti-CCP) in relation to* JAK3* gene polymorphisms. We performed this study in a subgroup of patients in whom carotid ultrasound and clinical and laboratory data were available. In this regard, no significant results were obtained in any of the analyses (Tables 5, 6, 7, 8, 9, and 10).

## 4. Discussion

CV disease is the main cause of death in patients with RA [4]. Therefore, a better understanding of the mechanisms involved in this disorder has become of main importance. During the last years, several genetic markers have been involved in CV disease susceptibility and progression in patients with RA [4–8].

JAK3 is a potential target for immunomodulatory drugs since it is involved in key inflammatory pathways in both autoimmune and CV diseases. In accordance, several pharmaceutical companies have reported JAK inhibitors in various stages of clinical development [28], and some clinical trials are ongoing to monitor the efficacy and safety of JAK3 inhibitor tofacitinib [29, 30].

*JAK3* polymorphisms have been associated with CV events in incident dialysis patients [19]. Because of that, in this study we analyzed two well-known polymorphisms rs3212780 and rs3212752 located in the* JAK3* gene. To the best of our knowledge, this is the first study performed to evaluate the potential influence of* JAK3 *polymorphisms in the risk of CV disease and subclinical atherosclerosis in an RA cohort. However, we did not observe any statistically significant differences when each polymorphism was assessed according to cIMT values and presence or absence of carotid plaques in RA. Besides an absence of association with subclinical atherosclerosis, we did not observe significant differences when RA patients were stratified according to the presence or absence of CV events. The discrepancy observed between our results and the ones obtained in incident dialysis patients [19] may be explained by the fact that both populations displayed very different characteristics. In this regard, and in contrast to the population described by Sperati et al. [19], the vast majority of our RA patients were not on dialysis due to end stage renal disease as the final stage of a chronic kidney disease. Additionally, the population assessed in that study was very heterogeneous, including both black and white individuals.

Nevertheless, even though our results are negative, we feel that these negative data are of potential interest and they may be of help to establish future lines of research. Further studies aimed at determining the potential influence of polymorphisms located in genes implicated in the inflammatory pathways on the risk of CV disease in RA are warranted.

## 5. Conclusion

Our results do not confirm association between* JAK3* polymorphisms and CV disease in RA.

## Figures and Tables

**Table 1:** Demographic characteristics of the RA patients.

Clinical features	% (*n*/*N*)
Patients	2136
Main characteristics	
Age at the time of disease onset (years, mean ± SD)	50.8 ± 14.8
Follow-up (years, mean ± SD)	11.6 ± 8.3
Percentage of women	75.2
Rheumatoid factor positive^*^	69.1 (1430/2071)
Anti-CCP antibodies positive	59.1 (1063/1799)
Shared epitope positive	62.6 (762/1217)
Erosions	55 (902/1640)
Extra-articular manifestations^**^	31.1 (511/1640)
Cardiovascular risk factors	
Hypertension	38.5 (810/2102)
Diabetes mellitus	12.4 (261/2102)
Dyslipidemia	36.0 (757/2102)
Obesity	18.1 (381/2102)
Smoking habit	24.5 (517/2102)
Patients with cardiovascular events	17.9 (384/2136)
Ischemic heart disease	8.4 (180/2136)
Heart failure	5.9 (126/2136)
Cerebrovascular accident	5.2 (112/2136)
Peripheral arteriopathy	2.4 (52/2136)

RA: rheumatoid arthritis; *n*: number of patients; SD: standard deviation; Anti-CCP antibodies: anti-cyclic citrullinated peptide antibodies.

^*^At least two determinations were required for analysis of this result.

^**^Extra-articular manifestations of the disease (if RA patients experienced at least one of the following manifestations: nodular disease, Felty's syndrome, pulmonary fibrosis, rheumatoid vasculitis, or secondary Sjögren's syndrome) [4].

**Table 2:** Differences in genotype and allele frequencies of *JAK3* polymorphisms between RA patients with or without cardiovascular (CV) events.

SNP	1/2	Subgroup	Genotype, *N* (%)				Allele test	
			1/1	1/2	2/2	MAF	*P* ^*^	OR [95% CI]^*^
rs3212780	G/**A**	Without CV events	909 (52.51)	688 (39.75)	134 (7.74)	0.28		
		With CV events	191 (50.26)	160 (42.11)	29 (7.63)	0.29	0.51	0.93 [0.75–1.06]

rs3212752	T/**C**	Without CV events	1547 (88.30)	203 (11.59)	2 (0.11)	0.06		
		With CV events	349 (90.88)	34 (8.85)	1 (0.26)	0.05	0.35	0.81 [0.52–1.26]

RA: rheumatoid arthritis. CV: cardiovascular. SNP: single nucleotide polymorphisms. MAF: minor allele frequency. OR: odds ratio. CI: confidence interval.

^*^Adjusted for sex, age at the time of ultrasonography study, follow-up time, and traditional CV risk factors (hypertension, diabetes mellitus, dyslipidemia, obesity, and smoking habit) by logistic regression.

**Table 3:** Association between *JAK3* polymorphisms and carotid intima-media thickness (cIMT) and presence/absence of carotid plaques in RA patients.

*JAK*3	Genotypes/alleles	cIMT mm		Presence versus absence of carotid plaques	
		Mean ± SD	*P* ^*^	OR [95% CI]^**^	*P* ^**^
rs3212780	GG (*n* = 285)	0.73 ± 0.17		Ref.	
	GA (*n* = 210)	0.73 ± 0.17		1.13 [0.79–1.61]	0.51
	AA (*n* = 44)	0.77 ± 0.22	0.38	1.54 [0.80–2.96]	0.20
	G (*n* = 780)	0.73 ± 0.17			
	A (*n* = 298)	0.74 ± 0.19	0.56	1.19 [0.91–1.56]	0.50

rs3212752	TT (*n* = 477)	0.73 ± 0.18		Ref.	
	TC (*n* = 60)	0.74 ± 0.17	0.17	0.61 [0.35–1.05]	0.15
	CC (*n* = 0)	—	—	—	—
	T (*n* = 1014)	0.74 ± 0.17			
	C (*n* = 60)	0.74 ± 0.17	0.17	0.62 [0.37–1.06]	0.15

RA: rheumatoid arthritis. cIMT: Carotid intima-media thickness. SD: standard deviation. OR: Odds Ratio. CI: confidence interval.

^*^Adjusted for sex, age at the time of ultrasonography study, follow-up time, and traditional CV risk factors (hypertension, diabetes mellitus, dyslipidemia, obesity, and smoking habit) using analysis of covariance (ANCOVA).

^**^Adjusted for sex, age at the time of ultrasonography study, follow-up time, and traditional CV risk factors (hypertension, diabetes mellitus, dyslipidemia, obesity, and smoking habit) by logistic regression.

**Table 4:** Association between *JAK3* polymorphisms and CRP levels in RA patients.

*JAK*3	Genotypes/alleles	CRP mg/L	*P* ^*^
		Mean ± SD	
rs3212780	GG (*n* = 311)	15.2 ± 25.9	0.58
	GA (*n* = 234)	13.5 ± 20.1	
	AA (*n* = 58)	17.8 ± 30.6	
	G (*n* = 856)	14.9 ± 24.4	0.99
	A (*n* = 350)	14.9 ± 24.1	
rs3212752	TT (*n* = 534)	14.7 ± 24.4	0.97
	TC (*n* = 72)	14.5 ± 23.7	
	CC (*n* = 2)	6.6 ± 7.2	
	T (*n* = 1140)	14.7 ± 24.3	0.95
	C (*n* = 76)	14.1 ± 23.2	

Haplotypes	GT (820)	14.7 ± 24.3	0.92
	AT (304)	15.4 ± 24.9	
	AC (41)	13.1 ± 17.7	
	GC (31)	16.7 ± 30.1	

CRP: C-Reactive Protein; RA: rheumatoid arthritis; SD: standard deviation.

^*^Adjusted for potential confounder factors.

**Table 5:** Association between cIMT values and *JAK3* polymorphisms in RA patients stratified according to anti-CCP status.

Subgroup	*JAK*3	Genotypes/alleles	cIMT (mm)	*P* ^*^
			Mean ± SD	
Anti-CCP positive	rs3212780	GG (*n* = 137)	0.73 ± 0.17	0.55
		GA (*n* = 97)	0.73 ± 0.18	
		AA (*n* = 21)	0.77 ± 0.23	
		G (*n* = 371)	0.73 ± 0.17	0.50
		A (*n* = 139)	0.74 ± 0.19	
	rs3212752	TT (*n* = 224)	0.73 ± 0.17	0.25
		TC (*n* = 31)	0.77 ± 0.18	
		CC (*n* = 0)	—	
		T (*n* = 479)	0.73 ± 0.18	0.27
		C (*n* = 31)	0.77 ± 0.18	
	Haplotypes	GT (*n* = 355)	0.73 ± 0.17	0.53
		AT (*n* = 124)	0.74 ± 0.19	
		AC (*n* = 15)	0.73 ± 0.21	
		GC (*n* = 16)	0.77 ± 0.17	

Anti-CCP negative	rs3212780	GG (*n* = 127)	0.71 ± 0.16	0.78
		GA (*n* = 102)	0.73 ± 0.17	
		AA (*n* = 22)	0.77 ± 0.19	
		G (*n* = 356)	0.72 ± 0.16	0.53
		A (*n* = 146)	0.74 ± 0.17	
	rs3212752	TT (*n* = 225)	0.72 ± 0.17	0.53
		TC (*n* = 24)	0.73 ± 0.15	
		CC (*n* = 0)	—	
		T (*n* = 474)	0.72 ± 0.17	0.54
		C (*n* = 24)	0.73 ± 0.15	
	Haplotypes	GT (*n* = 342)	0.72 ± 0.16	0.79
		AT (*n* = 132)	0.75 ± 0.18	
		AC (*n* = 14)	0.73 ± 0.17	
		GC (*n* = 10)	0.73 ± 0.11	

cIMT: carotid intima-media thickness; anti-CCP: anti-cyclic citrullinated peptide; RA: rheumatoid arthritis; SD: standard deviation.

^*^Adjusted for potential confounder factors.

**Table 6:** Association between cIMT values and *JAK3* polymorphisms in RA patients stratified according to RF status.

Subgroup	*JAK*3	Genotypes/alleles	cIMT (mm)	*P* ^*^
			Mean ± SD	
RF positive	rs3212780	GG (*n* = 192)	0.73 ± 0.16	0.41
		GA (*n* = 132)	0.73 ± 0.17	
		AA (*n* = 26)	0.79 ± 0.25	
		G (*n* = 516)	0.73 ± 0.16	0.42
		A (*n* = 184)	0.75 ± 0.19	
	rs3212752	TT (*n* = 312)	0.74 ± 0.17	0.52
		TC (*n* = 38)	0.73 ± 0.17	
		CC (*n* = 0)	—	
		T (*n* = 662)	0.73 ± 0.17	0.54
		C (*n* = 38)	0.73 ± 0.17	
	Haplotypes	GT (*n* = 498)	0.73 ± 0.16	0.67
		AT (*n* = 164)	0.75 ± 0.19	
		AC (*n* = 20)	0.72 ± 0.19	
		GC (*n* = 18)	0.74 ± 0.14	

RF negative	rs3212780	GG (*n* = 116)	0.73 ± 0.16	0.95
		GA (*n* = 93)	0.74 ± 0.17	
		AA (*n* = 20)	0.76 ± 0.15	
		G (*n* = 325)	0.73 ± 0.16	0.84
		A (*n* = 133)	0.74 ± 0.16	
	rs3212752	TT (*n* = 203)	0.73 ± 0.17	0.20
		TC (*n* = 24)	0.78 ± 0.16	
		CC (*n* = 0)	—	
		T (*n* = 430)	0.73 ± 0.17	0.21
		C (*n* = 24)	0.78 ± 0.16	
	Haplotypes	GT (*n* = 312)	0.73 ± 0.17	0.40
		AT (*n* = 118)	0.74 ± 0.17	
		AC (*n* = 15)	0.76 ± 0.16	
		GC (*n* = 9)	0.81 ± 0.16	

cIMT: carotid intima-media thickness; RA: rheumatoid arthritis; RF: rheumatoid factor; SD: standard deviation.

^*^Adjusted for potential confounder factors.

**Table 7:** Association between presence/absence of carotid plaques and *JAK3* polymorphisms in anti-CCP positive RA patients.

Subgroup	Presence of carotid plaques	*JAK*3	Genotypes/alleles	*n* (%)	Presence of carotid plaques	*JAK*3	Genotypes/alleles	*n* (%)	*P* ^*^	OR^*^[95% CI]
Anti-CCP positive	Yes	rs3212780	GG	76 (57.1)		rs3212780	GG	60 (49.6)	—	Ref.
			GA	44 (33.1)			GA	53 (43.8)	0.07	0.57 [0.14–1.03]
			AA	13 (9.8)			AA	8 (6.6)	0.72	1.21 [0.42–3.47]
			G	196 (73.7)			G	173 (71.5)	—	Ref.
			A	70 (26.3)			A	69 (28.5)	0.42	0.83 [0.53–1.29]
		rs3212752	TT	118 (88.7)		rs3212752	TT	104 (85.9)	—	Ref.
			TC	15 (11.3)			TC	17 (14.0)	0.36	0.67 [0.29–1.56]
			CC	—	No		CC	—	—	—
			T	251 (94.4)			T	225 (93.0)	—	Ref.
			C	15 (5.6)			C	17 (7.0)	0.38	0.69 [0.31–1.55]
		Haplotypes	GT	188 (70.1)		Haplotypes	GT	164 (67.8)	—	Ref.
			AT	63 (23.7)			AT	61 (25.2)	0.59	0.88 [0.55–1.40]
			AC	7 (2.6)			AC	8 (3.3)	0.24	0.49 [0.15–1.58]
			GC	8 (3.0)			GC	9 (3.7)	0.81	0.88 [0.29–2.59]

Anti-CCP: anti-cyclic citrullinated peptide; RA: rheumatoid arthritis; OR: odds ratio; CI: confidence interval.

^*^Adjusted for potential confounder factors.

**Table 8:** Association between presence/absence of carotid plaques and *JAK3* polymorphisms in anti-CCP negative RA patients.

Subgroup	Presence of carotid plaques	*JAK*3	Genotypes/alleles	*n* (%)	Presence of carotid plaques	*JAK*3	Genotypes/alleles	*n* (%)	*P* ^*^	OR^*^[95% CI]
Anti-CCP negative	Yes	rs3212780	GG	59 (45.7)		rs3212780	GG	69 (57.5)	—	Ref.
			GA	57 (44.2)			GA	43 (35.8)	0.20	1.50 [0.81–2.80]
			AA	13 (10.1)			AA	8 (6.7)	0.72	1.21 [0.41–3.65]
			G	175 (67.8)			G	181 (75.4)	—	Ref.
			A	83 (32.2)			A	59 (24.6)	0.32	1.26 [0.79–2.00]
		rs3212752	TT	119 (93.0)		rs3212752	TT	104 (87.4)	—	Ref.
			TC	9 (7.0)			TC	15 (12.6)	0.23	0.53 [0.19–1.47]
			CC	—	No		CC	—	—	—
			T	247 (96.5)			T	223 (93.7)	—	Ref.
			C	9 (3.5)			C	15 (6.3)	0.24	0.55 [0.21–1.48]
		Haplotypes	GT	169 (66.0)		Haplotypes	GT	173 (72.7)	—	Ref.
			AT	78 (30.0)			AT	50 (21.0)	0.16	1.42 [0.87–2.30]
			AC	5 (2.0)			AC	9 (3.8)	0.16	0.40 [0.11–1.45]
			GC	4 (1.6)			GC	6 (2.5)	0.93	1.06 [0.26–4.43]

Anti-CCP: anti-cyclic citrullinated peptide; RA: rheumatoid arthritis; OR: odds ratio; CI: confidence interval.

^*^Adjusted for potential confounder factors.

**Table 9:** Association between presence/absence of carotid plaques and *JAK3* polymorphisms in RF positive RA patients.

Subgroup	Presence of carotid plaques	*JAK*3	Genotypes/alleles	*n* (%)	Presence of carotid plaques	*JAK*3	Genotypes/alleles	*n* (%)	*P* ^*^	OR^*^[95% CI]
RF positive	Yes	rs3212780	GG	105 (57.1)		rs3212780	GG	86 (52.4)	—	Ref.
			GA	63 (34.2)			GA	68 (41.5)	0.08	0.64 [0.39–1.05]
			AA	16 (8.7)			AA	10 (6.1)	0.96	1.02 [0.41–2.56]
			G	273 (74.2)			G	240 (73.2)	—	Ref.
			A	95 (25.8)			A	88 (26.8)	0.30	0.82 [0.56–1.19]
		rs3212752	TT	170 (92.4)		rs3212752	TT	139 (84.8)	—	Ref.
			TC	14 (7.6)			TC	25 (15.2)	0.08	0.50 [0.24–1.07]
			CC	—	No		CC	—	—	—
			T	354 (96.2)			T	303 (92.4)	—	Ref
			C	14 (3.8)			C	25 (7.6)	0.09	0.52 [0.25–1.10]
		Haplotypes	GT	265 (72.0)		Haplotypes	GT	229 (69.8)	—	Ref.
			AT	89 (24.2)			AT	74 (22.6)	0.60	0.90 [0.61–1.33]
			AC	6 (1.6)			AC	14 (4.3)	0.06	0.33 [0.11–1.10]
			GC	8 (2.2)			GC	11 (3.4)	0.63	0.78 [0.28–2.15]

RF: rheumatoid factor; RA: rheumatoid arthritis; OR: odds ratio; CI: confidence interval.

^*^Adjusted for potential confounder factors.

**Table 10:** Association between presence/absence of carotid plaques and *JAK3* polymorphisms in RF negative RA patients.

Subgroup	Presence of carotid plaques	*JAK*3	Genotypes/alleles	*n* (%)	Presence of carotid plaques	*JAK*3	Genotypes/alleles	*n* (%)	*P* ^*^	OR^*^[95% CI]
RF negative	Yes	rs3212780	GG	57 (46.0)		rs3212780	GG	60 (57.7)	—	Ref.
			GA	55 (44.3)			GA	37 (35.6)	0.13	1.64 [0.86–3.14]
			AA	12 (9.7)			AA	7 (6.7)	0.58	1.39 [0.4–4.40]
			G	169 (68.1)			G	157 (75.5)	—	Ref.
			A	79 (31.9)			A	51 (24.5)	0.20	1.36 [0.84–2.22]
		rs3212752	TT	111 (90.2)		rs3212752	TT	91 (88.3)	—	Ref.
			TC	12 (9.8)			TC	12 (11.7)	0.45	0.69 [0.26–1.80]
			CC	—	No		CC	—	—	—
			T	234 (95.1)			T	194 (94.2)	—	Ref.
			C	12 (4.9)			C	12 (5.8)	0.46	0.71 [0.28–1.79]
		Haplotypes	GT	163 (66.3)		Haplotypes	GT	150 (72.8)	—	Ref.
			AT	71 (28.9)			AT	44 (21.4)	0.16	1.43 [0.86–2.40]
			AC	8 (3.3)			AC	7 (3.4)	0.71	0.79 [0.23–2.66]
			GC	4 (1.6)			GC	5 (2.4)	0.71	0.76 [0.18–3.14]

RF: rheumatoid factor; RA: rheumatoid arthritis; OR: odds ratio; CI: confidence interval.

^*^Adjusted for potential confounder factors.
